# Dermatology and Syphilis

**Published:** 1877-10

**Authors:** 


### I. Dermatology and Syphilis.

Psoriasis of the Hairy Scalp.—Lailler. (*La France Médicale.* Aug. 29, 1877. P. 546. No. 69.—Psoriasis occurring upon the scalp is characterized by the occurrence of patches of limited contour, clearly defined, and not discharging, as a rule, unless scratching has been induced by itching. Tne scalp is thickened, and the scales are dull and white in color and very adherent. In the large majority of cases there is no alopecia; the eruption often extends to the forehead, and sometimes to the ears.

Psoriasis of the scalp may be confounded with eczema, but in the latter disease, the eruption is more diffused, and the discharge is abundant. When eczema is in the pityriasic stage, the diagnosis must be established with caution. Apart from the aspect of the scaliness (in addition to other points of distinction which are to be remembered) it must be admitted that in certain cases it is difficult to pronounce as to the disease. The elbows and knees should be carefully inspected, and the diagnosis of psoriasis always be established, if in these localities are a few small patches to be discovered covered with white, dry and nacreous scales.

Impetigo can readily be distinguished from psoriasis by the color and character of its crusts, which are more elevated, yellowish and less dry. The presence of pustules is also significant.

As regards syphilis, when the crusts fall, ulcerations become visible, which are followed by cicatrices and alopecia.

When favus is distinctly present, there is no need of doubt, as the presence of the favus crusts is sufficient to clear up the case. But when the latter are absent, one might be disposed to call the disease psoriasis. In such cases, it should be remembered that the favus crusts are yellowish, the patch is more clearly defined and more reddened, the hairs are scanty, decolorized and rendered more like lanugo; cicatricial patches can often be seen.

In pityriasis, the eruption is diffused, does not present the elevated patches of psoriasis, the scalp is not thickened, and the scales are fine and furfuraceous.

Pityriasis rubra universalis. — Dr. Hans Hebra.— *Vierteljahresschr. f. Dermatol. & Syph.* III., 4).—In July, 1873, an Italian, aged 38 years, was admitted to the Vienna hospital, with pityriasis rubra universalis. He remained there three years and six months, and during this time, he was subjected to a number of therapeutic experiments, none of which, however, could make the slightest impression on the cutaneous disease.

When eight years old, the patient had the small-pox, after which his skin would always become exceedingly red in a warm temperature, and be very livid in cold air; it has never since been normal, but gradually acquired a deep red hue. Yet he was robust and could work, until two years previous to admission to the hospital. At that time he had to stop work, because the tension of the skin, which seemed contracted, impeded the free motions of the limbs. About that time he noticed the first formation of scales on his legs; eight months later, the whole body was covered with epidermic scales, and the hair of the scalp, whiskers and the pubes fell out. On admission, this was the patient's condition; the integument of the whole body was exceedingly tight, partly red, partly livid, the epidermis shedding in larger and smaller scales, in some places matted together, especially on the face. Both lower lids were everted by the shrinkage of the facial skin. Face and scalp were absolutely bald, and all over the body the growth of hair is greatly reduced. Patient is emaciated and feeble, though his appetite, digestion and sleep are good.

The only change which occurred in the patient's external appearance, while in the hospital, was a progressive discoloration of the skin, the red color passing over into a brown red tint, and an ultimate total alopecia.

In March, 1874, while the patient was laboring under an acute pneumonia, his integument improved remarkably; but as soon as he felt better, the cutaneous affection became again aggravated. He finally died of pulmonary and visceral tuberculosis, March 2, 1876, having spent 1,324 days in the hospital. During this time, he was the object of many experiments. Each remedy was tried one hundred days, unless an aggravation of the disease necessitated its entire discontinuance. Externally were used cod-liver oil, warm baths, benzoin, rubber cloth, ung. hydr. ox. albi. and ung. diachyli. The use of benzoin had to be stopped after three weeks, because it increased the dryness and tension of the skin to an unbearable degree. The other remedies had a soothing influence, making the skin softer and more pliable, bnt that was all. On the hyperæmia and desquamation their effect was nil.

Shortly after his admission the patient was dosed with arsenic. Taking one pill, containing 1-10 grain arsenic, three times daily, he swallowed 2,000 pills, or 200 grains of arsenic, in the year 1872, and two years later he went through the same course of treatment, so that in all he took 4000 *pills containing* 400 *grains of arsenic.* His appetite, digestion, and sleep were not disturbed by this treatment, but the pityriasis was not affected by it.

The autopsy confirmed the diagnosis of pulmonary and intestinal tuberculosis, but as to the cutaneous affection, it did not clear up its mysterious character.

The microscopic examination of pieces of the integument showed a chronic inflammatory infiltration of the skin. All its structures were infiltrated with cells in a state of rapid generation. Immediately under the very thick epidermis this proliferation was at its height; no sweat-gland or hair follicle could be detected in all the preparations. The whole picture gave the impression of a recent scar covered by epidermis.

WHITE SAND FOR SKIN DISEASES.—L. Ellinger. (*Wiener Med. Wochenschr.* 1876, No. 45.)—Acne, comedones, acne rosacea, freckles, prurigo, and other cutaneous diseases are treated by E. with fine white sand, such as is used for scrubbing floors. The sand must be of the right fineness, and not commingled with any coarse grains, nor too much dust. In facial eruptions the skin is washed with soap and water and kept constantly moistened for half an hour; then the affected spots are scrubbed with the moist sand, and the skin cleaned off again with a sponge or a brush. This procedure is repeated every evening.

In circumscribed cutaneous affections of the extremities, or the trunk, the affected part may be wrapped up in water dressing over night, the scrubbing to be done in the morning. If the disease extends over a larger surface, a full bath of one hour must precede the use of the sand.

The writer has been greatly pleased with the results of this treatment, the more so as he did not make any change in the patients' mode of living nor administer any medicine. He considers this method superior to the usual treatment of chronic skin diseases (with soft soap, tar, carbolic acid, etc.,) inasmuch as it has no disagreeable odor; does not keep the patient from business, and is a cheap and speedy cure.

Effect of Water on the Skin in Health and Disease.—Prof. Hebra.—(*Wiener Med. Worchenschr.* No. 132, 1877.) Prof. H., summing up his experience of the effect of the application of water upon the skin, arrives at the following conclusions: water exercises upon the skin a considerable degree of irritation, which may produce morbid symptoms, but also remove existing anomalies in the cutaneous tissues.

It is not the temperature but the macerating and irritating property of the water, that plays the principal part in its employment. As to the temperature of fomentations, ablutions, and baths, this should always be regulated by the feelings of the patient.

Ablutions of the whole body, or full baths—warm or cold—are entirely ineffective as a prophylactic measure against the outbreak of diseases of internal organs, but they often create diseases of the integument.

Baths taken for the treatment of cutaneous diseases must always be of a prolonged duration, in order to be useful; they should never be shorter than one hour at least; and warm baths may be continued without harm uninterruptedly day and night during several months.

Arterial Lesions Produced by Syphilis.—Lancereaux. (*Le Progrès Med.*, *Sept.* 1, 1877, *No.* 35, *p.* 676).—Such lesions are much more common than is generally believed to be the case, and they present the peculiarity of affecting especially the encephalic arteries. The sites of predilection are the vertebral arteries, the basilar axis, and the sylvian arteries, and the anatomical feature of these syphilitic alterations, is their circumscription. Thus the islets of arteritis scarcely measure more than one to ten centimetres in length, and rarely attain that dimension. The commencement of the process is in the internal tunic of the arteries, non-vascular tissue. There a slight elevation occurs, a species of pustule forms like the primary patch of atheroma. When these little tumors open into the lumen of the vessel, cavities result which become small aneurisms. (Several plates portraying these lesions were exhibited to the Medical Section of the *Association francaise pour l'avancement des Sciences* by M. Lancereaux.)

There are cases where these aneurisms undergo a species of development; in others, a real arterial obliteration occurs. How are these lesions to be distinguished from atheroma, which they so much resemble? Several points of distinction are given. 1. The subject is syphilitic. 2. Atheroma is specially developed in the large arteries, the aorta, the splenic mesenteric and renal arteries, while the alterations produced by syphilitic arteritis affect chiefly the arteries of the encephalon. 3. The subject is, as a rule, young, while atheroma is a disease of the aged. 4. Finally, it is not rare to note a certain degree of symmetry in the lesions. The author concludes with a study of the symptoms of syphilitic arteritis, and the means by which it can be clinically recognized from atheroma and embolism. In embolism the phenomena are immediate; in syphilitic arteritis they are slow of development and preceded by prodromata, cephalalgia and insomnia. These phenomena, it is true, also accompany atheroma, but here the age of the patient is an aid in coming to a decision. Since atheroma is a disease of the aged, and syphilitic arteritis of the young, it becomes highly probable that when the encephalic arteries are affected with atheroma, those of the limbs will be also. Lancereaux claims that he has made a diagnosis of syphilitic arteritis in the living by these symptoms alone. The disorder is grave, the prognosis serious. In the emergency no delay should be had: large doses of iodide of potassium and abundant mercurial inunctions should be employed.

The Abortive Treatment of Syphilis.—G. E. Weisflog. (*Virchow's Arch. Bd.* 69; *Allg. Med. Centralztg*).—A few years ago, W. discovered the remarkable fact that a watery solution of the hydrargyrum nitricum oxydulatum injected under the skin never causes suppuration unless the tissues are in an inflamed state; but where the tissues are in a state of irritation or inflammation, an abscess will always follow such hypodermic injection. This property of the hydrarg. nitric. oxydulatum can be successfully employed to check the propagation of the syphilitic virus on its way from the original primary sore to the inguinal glands. In all suspicious cases, therefore, he begins the local treatment with an hypodermic injection into the region between the genitals and the inguinal glands. If these, or the lymphatic vessels leading to them, are not already in a state of irritation or inflammation, no suppuration will take place; nevertheless no constitutional syphilis will follow, provided that the subcutaneous injections be repeated every tenth day until the primary ulcer is perfectly cured and its induration completely removed. But if at the time the injection is made, the lymphatic vessels or glands are already in a state of irritation or inflammation, an abscess will be formed whose contents have a characteristic chocolate color. These abscesses very seldom give rise to any violent symptoms, and heal very quickly. They have never been followed by constitutional syphilis.

He has practiced this abortive treatment the past five years, and from 1820 to 1872 treated 32 cases of undoubted indurated chancre; the injections caused the formation of an abscess on both sides in 14 cases, and on one side in six cases. Though at that time he felt positively certain that his patients had nothing to apprehend in the future, he has tried, within the last six months, to ascertain, by letter or personal interview, whether the expectations had been verified. He obtained news from 28 former patients—they all remained free from any affection which could be recognized as a sequela of the local infection; twelve of them had married, and their offspring did not show any symptoms of inherited syphilis.

Although the author is willing to admit that there is no conclusive proof in so small a number of cases, he is confident that a conscientious trial by others will confirm his experience. Mercury is most efficient as an anti-syphilitic, if brought in direct contact with the virus in the affected tissues; and the hypodermic injection is the simplest method of saturating with the metal those tissues which the syphilitic virus must pass, on its way from the chancre to the glands. It is very probable, therefore, that other remedies known to antagonize the virus of the primary ulcer may be employed for the hypodermic treatment with success; but the author had no occasion for experimenting on this point, inasmuch as the watery solution of the hydrarg. nitric. oxydulatum possessed every property that could be desired for the purpose. It is a mild and durable solution.

Syphilis Treated by Subcutaneous Injections of Calomel.—Kœlliker. (*Centralbl. f. Chir.* 1877, *p.* 97).—During 1875 and 1876 the syphilitic patients in the hospital at Wurzburg were treated by hypodermic injections of calomel (3,0) suspended in glycerine (30,0). The dose of calomel injected was 0,05 for an adult, and 0,025 for children. The injections, made with an ordinary hypodermic syringe, were repeated every tenth day at first; later, every fifth day. The mode of treatment is easy, does not disturb the nutrition of the patient, and permits of the dose of mercury administered being accurately measured. But it shares with other hypodermic methods the unpleasant feature of the very frequent occurrence of abscesses at the punctures.

